# Ebselen, a promising host-directed therapeutic option against *Yersinia pseudotuberculosis* infection

**DOI:** 10.3389/fmicb.2026.1749626

**Published:** 2026-02-11

**Authors:** Zhongbao Wu, Lu Tang, Yueqing Wang, Emmanuelle Vladia Ndong Ella, Jing He, Chuang Cheng, Lin Xiao, Yalan Wang, Chuanjiang Dong, Lili Zou, Jun Wang

**Affiliations:** 1 Hubei Clinical Research Center for Precision Prevention and Treatment of Gastrointestinal Cancer in Elderly People, The Second People’s Hospital of China Three Gorges University, Yichang, Hubei, China; 2 Key Laboratory of Tumor Microenvironment and Immunotherapy and Yichang Key Laboratory of Infection and Inflammation, College of Basic Medical Sciences, China Three Gorges University, Yichang, Hubei, China; 3 Yiling Maternal and Child Health Hospital, Yichang, Hubei, China; 4 Department of Urology, The First Dongguan Affiliated Hospital, Guangdong Medical University, Dongguan, Guangdong, China

**Keywords:** *Yersinia pseudotuberculosis*, ebselen, [host-directed therapy], macrophage polarization, antioxidant

## Abstract

**Background:**

As a prevalent food-borne zoonotic pathogen, *Yersinia pseudotuberculosis* (*Y. pseudotuberculosis*) can lead to severe health issues in both animals and humans. At present, therapeutic options are quite limited. This study evaluated the pharmacological properties of ebselen (EbSe) and its potential as a host-directed therapy (HDT) against *Y. pseudotuberculosis* infection. EbSe has shown efficacy and safety in clinical settings, particularly against Gram-positive bacteria, as evidenced by its pharmacological properties and clinical applications; however, its efficacy against Gram-negative bacteria remains poorly characterized.

**Methods:**

To systematically elucidate the mechanism of Ebse efficacy, an in vivo mouse model of acute gastroenteritis induced by Y. pseudotuberculosis and an in vitro macrophage-bacteria interaction model were established. Monitoring mouse survival rates and bioluminescence imaging analysis revealed Ebse’s ability against *Y. pseudotuberculosis* infection. Flow cytometry characterized peritoneal macrophage polarization types and in vitro ones. Transcriptome analysis identified differentially expressed mRNAs in macrophages post-*Y.pseudotuberculosis* infection, validated by real-time quantitative PCR (RT-qPCR). Cell death was assessed using the CCK-8 assay and propidium iodide (PI) staining, supplemented by morphological observation via transmission electron microscopy (TEM). The intracellular bacteria were quantified through Cytation 5 imaging and fluorescent quantification. Cellular reactive oxygen species (ROS) production was measured by flow cytometry, while glutathione (GSH) content was determined using a micro-reduction GSH assay kit. Intracellular thioredoxin reductase 1 (Txnrd1) activity was assessed via the 5,5’-dithiobis(2-nitrobenzoic acid) (DTNB) assay, with protein expression levels detected by Western blot.

**Results and discussion:**

EbSe improved overall survival and reduced bacterial burden in mice with gastroenteritis. EbSe was found to modulate macrophage polarization, inhibiting apoptosis and excessive inflammation, as detailed in macrophage function and their role in inflammatory responses. Furthermore, EbSe has been shown to protect Raw264.7 cells from *Y. pseudotuberculosis*-induced death by enhancing antioxidant defenses. This is evidenced by a reduction in intracellular reactive oxygen species levels and an elevation in glutathione concentrations, and increased thioredoxin reductase 1 activity. Collectively, these findings suggest that EbSe enhances the host resistance to *Y. pseudotuberculosis* infection, likely through immunomodulatory effects rather than direct antibacterial activity against Gram-negative bacteria.

## 1 Background

*Yersinia pseudotuberculosis* (*Y. pseudotuberculosis*), a significant food-borne zoonotic pathogen within the Enterobacteriaceae family, ranks as the third most common cause of bacterial enteritis of food-borne origin in Europe (Bai et al., 2020). *Y. pseudotuberculosis*, transmitted via contaminated food and water, causes gastroenteritis in humans and a variety of domestic and wild animals, including swine, sheep, deer, cattle, and poultry such as turkeys, ducks, and geese (Hulinova Stromerova and Faldyna, 2018; Rattner et al., 2024; Slee and Button, 1990; Riede et al., 2025). Systematic *Y. pseudotuberculosis* infection can severely impact the digestive tract, lymph nodes, and the respiratory system. Generally, prompt initiation of antimicrobial therapy is recommended to treat the *Y. pseudotuberculosis*, with doxycycline combined with gentamicin or ciprofloxacin/ ofloxacin (Tan et al., 2025) as the preferred regimen. However, numerous studies have documented that the misuse of antibiotics has resulted in alarmingly high resistance rates (Willcocks et al., 2019; Grygiel-Górniak, 2025). Furthermore, its ability to grow at both 4 and 25 °C enables prolonged environmental persistence (Fukushima et al., 2011), posing a continuous threat to animal health. Therefore, novel therapeutic strategies are urgently needed to mitigate the infection risks that *Y. pseudotuberculosis* poses to both human and animal populations.

The innate immune system, particularly macrophages, play a critical role in defending against *Y. pseudotuberculosis* infection. Macrophages recognize, phagocytose, and eliminate bacteria through mechanisms, including reactive oxygen species (ROS)- and reactive nitrogen species (RNS)-dependent killing, as well as lysosomal degradation (Català et al., 2022; Dramé et al., 2020; Weiss and Schaible, 2015). The oxidative stress status of the host is defined by the imbalance between ROS production and the antioxidant defense system (e.g., glutathione, thioredoxin), which plays a crucial role in regulating the antimicrobial function of macrophages (Herb and Schramm, 2021). Moderate oxidative stress boosts macrophage-mediated pathogen clearance by activating the NADPH oxidase (NOX) complex to produce ROS, enhancing phagocytic activity, and regulating pro-inflammatory signaling pathways (Li et al., 2025). In contrast, excessive oxidative stress or compromised antioxidant defense could severely damage macrophage viability, thereby promoting bacterial survival and dissemination (Hu et al., 2024). Thus, the oxidative stress status of the host serves as a key regulator of macrophage function, and targeting this balance represents a promising host-directed therapy (HDT) strategy. Rather than directly targeting pathogens, HDT therapy enhances the host’s resistance to infection and alleviates infection-induced pathological damage by modulating the host’s physiological status, immune responses, and tissue microenvironment. Compared with traditional antimicrobials, its core advantages include mitigating the selective pressure for antimicrobial resistance, regulating immune homeostasis, and preserving the host’s normal microbiota (Bergman et al., 2020).

Excessive macrophage apoptosis and inflammation are major contributors to the severity of *Y. pseudotuberculosis*-induced disease, indicating that mitigating macrophage cell death may enhance the host defense against this pathogen (Chow et al., 2016). Hence, to deal with infections in which *Y. pseudotuberculosis* subverts immune clearance, disrupts cellular redox homeostasis, and induces inflammatory cell death (Avican et al., 2017; Fasciano et al., 2021; Kobayashi et al., 2018; Ruckdeschel, 2002), the HDT strategy aimed at enhancing host immunity represents a highly promising option.

Previous studies from our group established that ebselen (EbSe), a synthetic organoselenium compund, exhibited potent direct bactericidal activity against Gram-positive bacteria (e.g., *Staphylococcus aureus*; Chao et al., 2022) but lacked efficacy against Gram-negative pathogens (e.g., *Escherichia coli, Acinetobacter baumannii*; Dong et al., 2020; Wang P. et al., 2020). Notably, preliminary findings demonstrated that EbSe significantly improved overall survival in murine gastroenteritis (Dong et al., 2022), despite its limited antimicrobial efficacy against Gram-negative bacteria *in vitro*. This apparent contradiction prompted further investigation.

To elucidate the HDT mechanism underlying EbSe’s protective effects against *Y. pseudotuberculosis* infection, we employed a *Y. pseudotuberculosis*-macrophage interaction model. Macrophages were infected with the *Y. pseudotuberculosis* YpIII strain and subsequently treated with EbSe. Both *in vivo* murine models and *in vitro* macrophage infection assays were conducted to elucidate the mechanistic basis underlying EbSe’s efficacy.

## 2 Materials and methods

### 2.1 Materials

#### 2.1.1 Experimental cell lines

Mouse mononuclear macrophage leukemia cells (Raw264.7) were acquired from Wuhan Punosai Life Technology Co., Ltd.

#### 2.1.2 Experimental strains

The *Y. pseudotuberculosis* YpIII strains was obtained from Uppsala University, Sweden. YpIII strains with different reporter systems on the plasmid were constructed and listed in Table 1. All experiments were carried out in the BSL-2 laboratory.

**Table 1:** *Yersinia pseudotuberculosis* YpIII strains with different reporter systems.

Strain	Genotype	Experiment
YpIII-GFP	*Yersinia pseudotuberculosis* (pCD1 expressing GFP, KM^r^)	*in vitro*
YpIII-bioluminescent	*Yersinia pseudotuberculosis*, Xen4 (pCD1 With Tn1000: Tn5 luxCDABE, KM^r^)	*in vivo*

#### 2.1.3 Experimental animals

A total of 96 male Kunming mice (specific pathogen free, SPF grade) were acquired from the Laboratory Animal Center of China Three Gorges University and housed individually in separate cages. All mice had an initial body weight ranging from 18 to 20 g. The animals were acclimatized for 1 week prior to the experiment.

##### 2.1.3.1 Housing conditions and experimental ethics

Housing environment control: The temperature was maintained at (22 ± 2) °C, with relative humidity at (60 ± 5)%, and a 12 h light/ 12 h dark cycle was set. During the housing period, mice had free access to standard feed and sterile water.

Experimental ethics approval: All animal experiments in this study were conducted in strict compliance with the ‘Guidelines for the Care and Use of Laboratory Animals’, adhering to the principles of laboratory animal welfare and ethics as outlined in the GB/T 35892–2018 ‘Laboratory Animal - Guideline for Ethical Review of Animal Welfare’. The animal experiments conducted at China Three Gorges University have been approved by the China Three Gorges University Medical Animal Care and Welfare Committee, ensuring compliance with ethical standards and scientific rigor (Approval No. 2023020E).

### 2.2 Main reagents

Luria-Bertani (LB) medium (EMD Millipore), 2-phenyl-1, 2-benzisoselenazol-3 (2H)-one (EbSe) (Selleck), Hyclone DMEM (Cytiva), Fetal bovine serum (Gibco), Gentamicin sulfate (Sangon Biotech, A506614), 2 × Universal SYBR Green Fast qPCR Mix (ABclonal), SweScript RT II First Strand cDNA Synthesis Kit (Service), Brilliant Violet 421^™^ anti-mouse (CD68) (BioLegend), Brilliant violet 510 nm anti-mouse (CD86) (Biolegend), PE/Cyanine7 anti-mouse CD206 (MMR) (Biolegend), Purified anti-mouse (CD16/32) (Biolegend), CellROX™ Deep Red Reagent (Invitrogen), Anti-Actin antibody (Proteintech), Anti-Txnrd1 antibody (Proteintech), Protein inhibitor cocktail (MedChemExpress), RIPA Lysis Buffer (Applygen).

#### 2.2.1 Ebse solution preparation

##### 2.2.1.1 For cell experiments

Based on EbSe’s molar mass (274.176 g/mol), add an appropriate volume of dimethyl sulfoxide (DMSO) to prepare a 100 mM stock solution. Thoroughly mix the solution by pipetting it up and down repeatedly. Prepare and store the stock solution in a light - protected environment.

##### 2.2.1.2 For *in vivo* experiments

Dissolve EbSe in DMSO to prepare a 100 mg/mL stock solution. Prepare the 4 mg/mL working solution by mixing the EbSe stock solution, PEG300, 5% Tween 80, and 50% ddH_2_O in a volume ratio of 5:40:5:50. Add the reagents one by one in sequence, and thoroughly mix the solution after adding each reagent addition until the solution is clear and transparent. Prepare the working solution fresh for each use and store all solutions protected from light.

### 2.3 Experimental methods

#### 2.3.1 Acute mouse *Yersinia pseudotuberculosis* gastroenteritis model

Forty-eight healthy male Kunming mice were randomly divided into 4 groups (*n* = 12). The infected group was gavaged with 100 μL 2 × 10^9^ CFU *Y. pseudotuberculosis* YpIII-bioluminescent, and intraperitoneally injected with 20 mg/Kg EbSe (Dong et al., 2022) and DMSO at 1, and 3 days post-infection to have groups A (DMSO), B (EbSe), C (YpIII + DMSO), and D (YpIII + EbSe). Group C and D were used for overall survival observations. Groups A-D were performed bioluminescence-monitoring at 1-, 4-, and 7-days post-infection for bacterial load detection. The *in vivo* imaging was conducted blindedly.

Another 48 mice were constructed for *Y. pseudotuberculosis*-YpIII gastroenteritis model as described above, solely used for peritoneal macrophage extraction in 2.3.4.

#### 2.3.2 Mice anesthesia/euthanasia methods

##### 2.3.2.1 Method of anesthesia for mice (IsoFluVet inhalation)

To analyze bacterial load in the gastrointestinal tracts, mice were intragastrically administered with *Y. pseudotuberculosis* YpIII-bioluminescent, anesthetized using the XGI-8 gas anesthesia system with 2.5% IsoFluVet in oxygen, and imaged by bioluminescent imaging. The entire procedure adhered to ‘the 2020 AVMA Guidelines’ for the use of IsoFluVet inhalation.

##### 2.3.2.2 Method of euthanasia for mice (carbon dioxide inhalation)

After overall survival analysis, mice were euthanized by gradual exposure to carbon dioxide (CO_2_) in strict accordance with ‘the 2020 AVMA guidelines’ for the euthanasia of animals. Mice were individually placed into a clean, transparent euthanasia chamber pre-filled with room air. CO_2_ (medical grade) was introduced at a displacement rate of 30–70% of the chamber volume per minute until irreversible loss of the confirmation of loss of consciousness, cessation of breathing, and heartbeat was made.

All protocols received approval from the China Three Gorges University Medical Animal Care and Welfare Committee and strictly complied with ‘the ARRIVE 2.0 guidelines’.

#### 2.3.3 Bioluminescence imaging analysis

Before *in vivo* imaging, the mice were fasted and deprived of water for 16 h. Bioluminescence was monitored using the IVIS Lumina II imaging system (Caliper Life Sciences) at 1-, 4-, and 7- days post-infection. Prior to imaging, the mice were anesthetized using the XGI-8 gas anesthesia system (Caliper LifeSciences) with 2.5% IsoFluVet in oxygen (Orion Pharma Abbott Laboratories Ltd., Great Britain), and 0.5% IsoFluVet was used during imaging. Images were collected and analyzed using Living Image 4.5 (Caliper LifeSciences).

**Figure 1:**
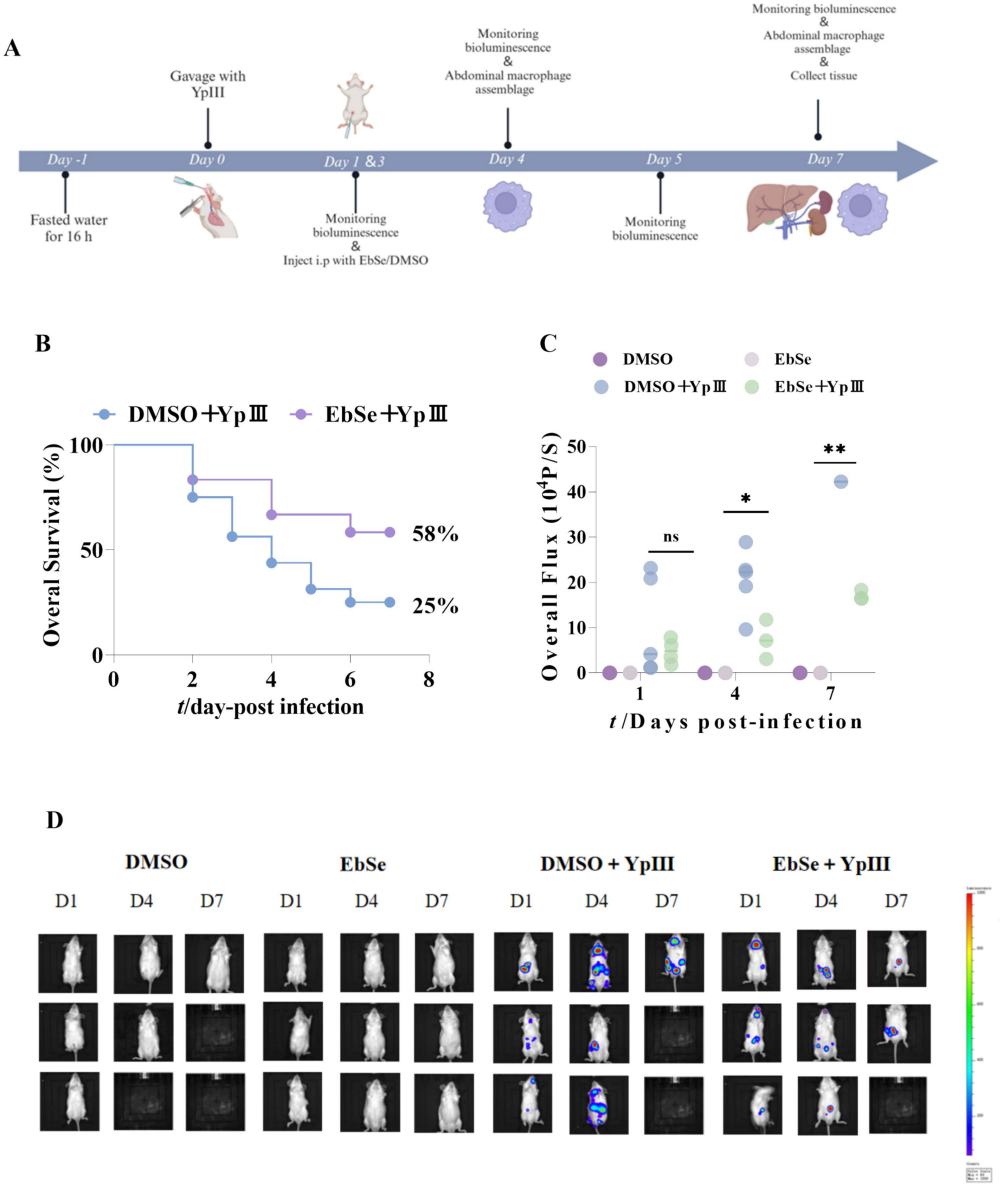
EbSe protects mice from *Y. pseudotuberculosis* YpIII-caused gastroenteritis. Mice were given *Y. pseudotuberculosis* YpIII via gavage, and then EbSe was i.p. administered; **(A)** Diagram demonstrating the animal infection model’s pattern; **(B)** Overall survival was observed for 7 days post-infection (*n* = 12). The Ebse+YpIII group is 58% (95% CI 28–84%), 2.3 folds relative to the DMSO+YpIII group (95% CI 5–57%); **(C,D)** Bioluminescence emission from the same mice was observed using IVIS lumina II (Caliper LifeSciences) on days 1, 4, and 7 post-infection, and the total luminous flux in each group of surviving mice was quantified. *p*-values of < 0.05 were significant. *p < 0.05; **p < 0.01; ***p < 0.001.

This analysis determined bacterial load by measuring *in vivo* bioluminescence intensity in mice at 1, 4, and 7 days post-infection. To ensure image clarity, each row in the Figure 1D represents imaging data from the same mouse at different time points.

#### 2.3.4 Extraction of mouse peritoneal macrophages

First, the mice were euthanized, and were submerged in 75% ethanol for 1 min. Then, sterile scissors were used to make a longitudinal incision in their abdomens, and a 5-mL disposable syringe was employed to inject 4 mL of DMEM culture medium into the abdominal cavity through the peritoneum beneath the cardiac fossa. Next, a sterile cotton swab was used to gently massage the entire abdominal cavity, ensuring that the macrophages were thoroughly rinsed with sterile water. Subsequently, a syringe was used to aspirate the culture medium, and the extracted cells were transferred to centrifuge tubes and temporarily stored on ice.

The isolated macrophages were centrifuged at 1,500 rpm for 10 min. Subsequently, if a red precipitate was observed post-centrifugation, erythrocyte lysis was initiated: the supernatant was discarded first, and then 2 mL of erythrocyte lysis buffer was added to resuspend the cells. After that, the cell suspension was incubated in a 37 °C water bath for 5 min to induce lysis, and then a second centrifugation at 1,500 rpm for 10 min was carried out. If residual red precipitate remained, the lysis procedure described above was repeated until complete lysis of all erythrocytes was achieved. After erythrocyte lysis, the isolated mouse peritoneal macrophages were employed for subsequent experiments.

#### 2.3.5 Immunofluorescence experiment

After cell collection, Fc receptors were blocked by incubating the cells with CD16/32 antibody at room temperature for 30 min. Unbound antibodies were removed by washing with PBS and centrifugation at 800 rpm for 10 min, followed by discarding the supernatant. Subsequently, cells were stained with BV421-conjugated anti-CD68 and BV510-conjugated anti-CD86 antibodies at 4 °C in the dark for 40 min, and then subjected to additional PBS washes and centrifugation (800 rpm, 10 min). For intracellular staining, cells were fixed and permeabilized with 100 μL of flow cytometry fixation/permeabilization solution at 4 °C in the dark for 30 min. After washing with 1 × permeabilization buffer, cells were incubated with PE/ Cy7-conjugated anti-CD206 antibody for 40 min at 4 °C in the dark. Finally, the cells were washed with PBS, resuspended in 300 μL of PBS, and then analyzed by flow cytometry.

#### 2.3.6 Hematoxylin and eosin staining

Liver and kidney tissues were collected and fixed in 4% paraformaldehyde (PFA) for 24–48 h. Fixed tissues were rinsed with PBS, dehydrated through a graded ethanol series (70, 80, 90, 100%; 1–2 h per step), cleared in xylene, and embedded in paraffin. Blocks were sectioned at 3–5 μm thickness using a microtome, and sections were floated on a water bath, mounted on glass slides, and dried overnight at 37 °C. For histological analysis, sections were deparaffinized, rehydrated, and stained with H&E using standard protocols. Nuclei were stained with hematoxylin, differentiated in acid alcohol, blued in ammonia water, and counterstained with eosin.

Finally, slides were dehydrated, cleared in xylene, and mounted for microscopic examination. After tissue staining was completed, CaseViewer software was used to record the scanned images. Histological grading has been carried out, and the procedure was performed blindly.

#### 2.3.7 Transcriptomics analysis (RNA-sequencing)

##### 2.3.7.1 Sample preparation and grouping

The Raw264.7 cells were subjected to transcriptomic sequencing (RNA-seq) analysis 24 h post infection with *Y. pseudotuberculosis* YpIII-GFP with the multiplicity of infection (MOI) of 50. Primarily, RNA-seq was performed in two groups: uninfected and infected cells treated by 20 mg/kg DMSO or EbSe.

##### 2.3.7.2 Experimental procedure and differential expression analysis

The RNA extraction and sequencing were employed by Novogene Technology Co., Ltd. (Beijing, China). Totally, in the uninfected group, EbSe induced differential expressions in 46 genes, whereas altered the expression of 3,223 genes in the YpIII-infected group. Enrichment analysis revealed that 161 and 20 of these genes were associated with inflammatory responses and immune regulation, respectively, motivating subsequent investigation into EbSe’s mechanisms.

#### 2.3.8 Determination of cell proliferation and cytotoxicity

The Raw264.7 cell suspension was diluted to a concentration of 1 × 10^5^ cells/mL, and 100 μL of the diluted suspension was added to each well of 96-well cell culture plates. Once the cells had adhered, the culture medium was removed, and the cells were rinsed three times with PBS.

Subsequently, Raw264.7 cells were infected with a pre-determined quantity of infection complexes for 1 h. For the infected group, extracellular bacteria were treated with gentamicin (60 μg/mL) for 1 h. Post-infection, the cells were divided into 2 groups, with 20 μM EbSe or DMSO respectively, followed by incubation for 24 h. Each group was set up with four biological replicates.

Following the incubation period, the cells were subjected to three PBS washes to remove any unbound substances. Subsequently, 10 μL of CCK-8 solution was introduced into each well and incubated for 2 h. The absorbance at 450 nm was then measured using a microplate reader to determine the cell viability based on the A_450_ values.

#### 2.3.9 Cell death assay

*Yersinia pseudotuberculosis* YpIII-infected Raw264.7 cells (5 × 10^6^) were collected and washed with PBS three times, followed by centrifugation at 800 rpm for 3 min. The cell pellet was gently resuspended in 500 μL of binding buffer to create a single-cell suspension. Subsequently, 5 μL of Propidium Iodide (PI) was added to the suspension and thoroughly mixed.

After incubation for 10 min at room temperature in the dark, the cells were observed and detected using a flow cytometer (Beckman Coulter, AW15093).

#### 2.3.10 Intracellular bacteria determination

After washing the cells three times with PBS, *Y. pseudotuberculosis* YpIII-infected Raw264.7 cells (5 × 10^6^) were collected using a cell scraper. The cells were gently resuspended in 300 μL of PBS to form a single-cell suspension. Due to the GFP tag on YpIII, the samples were directly analyzed through Cytation 5 imaging and fluorescent quantification.

#### 2.3.11 Cell morphology observation by transmission electron microscopy

*Yersinia pseudotuberculosis* YpIII-infected Raw264.7 cells (1 × 10^6^) were collected and subjected to centrifugation at 800 rpm for 3 min and subsequently fixed with 2.5% glutaraldehyde. The cell morphology was meticulously observed using a transmission electron microscope (TEM, Hitachi H-7500), following standard protocols to ensure the integrity of ultrastructural details.

#### 2.3.12 Real-time fluorescence quantitative PCR

Raw264.7 cells (1 × 10^7^) infected with *Y. pseudotuberculosis* YpIII for 0, 2, 4, 8, 16 and 24 h were collected, respectively. Total RNA was extracted by Trizol method, and reverse transcription was performed according to the instructions of SweScript RT II First Strand cDNA Synthesis Kit. RT-qPCR was conducted following the detailed instructions for the 2 × Universal SYBR Green Fast qPCR Mix, with GAPDH serving as the internal reference. This involved careful preparation of the reaction mix, adherence to specific experimental protocols, and the inclusion of appropriate controls to ensure the accuracy and reliability of the results. The detailed information on the amplification of the target gene is presented in Table 2, which is a crucial component of the data analysis.

**Table 2:** Sequences of primers used for RT-qPCR.

Gene	Sequence (5′-3′)
*gapdh*	F: TCTCCTGCGACTTCAACA R: TGTAGCCGTATTCATTGTCA
*inos*	F: TACTGCTGGTGGTGACAA R: CTGAAGGTGTGGTTGAGTT
*il-6*	F: TGGTCTTCTGGAGTACCATAGC R: TGTGACTCCAGCTTATCTCTTGG

#### 2.3.13 Intracellular ROS assay

Raw264.7 cells (5 × 10^6^) infected with *Y. pseudotuberculosis* YpIII for 24 h were collected, rinsed three times with PBS, and gently detached using a cell scraper, followed by centrifugation (800 rpm for 3 min) to remove the supernatant. The CellROX™ Red reagent was diluted at a ratio of 1:1000 in PBS and then added to the cells. Subsequently, the cells were stained in a 37 °C incubator for 30 min. When the incubation reached the 15 min mark, the tubes were gently inverted once to ensure homogeneity. Upon completion of the 30-min-incubation, unbound probes were removed by washing the cells three times with PBS. Cellular ROS production was quantified using flow cytometry, which involves the measurement of ROS levels within cells through the use of fluorescent probes and high-throughput analysis. The experiment should be completed within 30 min to prevent fluorescence quenching.

#### 2.3.14 Intracellular glutathione assay

After the protein of Raw264.7 cells (5 × 10^6^) infected with *Y. pseudotuberculosis* YpIII was extracted, the micro-reduced GSH assay kit was used for operation, and A_405_ was measured by an enzyme-labeled instrument for recording and data in process. The data of the GSH amount assays were normalized per cell concentration.

#### 2.3.15 Determination of intracellular Txnrd1 activity

The protein solution with 25 μg protein content in each group was filled with ultra-pure water to 20 μL and then mixed with 50 mM Tris–HCl (pH = 7.5), 1 mM EDTA, 5 μM Trx, 1 mM DTNB, 200 μM NADPH. In the first 30 min, the activity of Txnrd1 was measured at A_412_ by using the slope of the first 5 min.

#### 2.3.16 Determination of Txnrd1 protein in cells

Lysis Buffer and Cysteine Protease inhibitor were added to the RIPA mixture. After being rested on ice for 15 min, the supernatant was obtained by centrifugation (4 °C, 1200 rpm, 10 min), and then immunoblotting was carried out using an anti-Txnrd1 polyclonal antibody (Proteintech).

#### 2.3.17 Data statistics and analysis

Statistical analyses were performed using the comprehensive suite of statistical tools available in GraphPad Prism 9.5 (GraphPad Software). Each set of experiments was independently repeated three times or more. All data distribution were tested for normality and *post hoc* tests were used for multiple comparisons if applicable. Means of data between the two groups were contrasted using an unpaired Student’s *t*-test. Kaplan–Meier analysis and log-rank tests were used for survival data. Sample rates between the two groups were tested with Chi-square analysis. *p*-values of < 0.05 were significant.

## 3 Results

### 3.1 EbSe protects mice from *Yersinia pseudotuberculosis* YpIII-caused gastroenteritis

Recognized as the most common infection brought on by *Y. pseudotuberculosis* YpIII, gastroenteritis is fatal and hard to treat (Wang et al., 2016). To evaluate whether EbSe could protect mice from *Y. pseudotuberculosis* YpIII-induced gastroenteritis, 24 mice were randomly divided into two groups and gavaged with 2 × 10^9^ CFU/100 μL of YpIII-bioluminescent to establish acute gastroenteritis models (Figure 1A). Mice were further intraperitoneally injected with 20 mg/kg of Ebse on days 1- and 3-post-infection. On day 7 post-infection, the survival rate of mice treated with EbSe was significantly higher at 58% compared to the control group at 25% (Figure 1B; *p* < 0.05).

To further analyze the effect of EbSe, mice were monitored for *in vivo* imaging studies and quantitative data on bioluminescence values on days 1-, 4-, and 7-post-infection (Figure 1A). The results showed that the EbSe-treated group exhibited the lowest total luminous flux values, indicating the lowest bacterial load (Figures 1C,D). Hematoxylin and eosin (H&E) staining of the tissues (livers and kidneys) were performed on 7 days post-infection, revealing that EbSe led to a reduction in the systematic inflammation (Supplementary Figure S1). All the above results demonstrated that EbSe was able to protect mice from gastroenteritis caused by *Y. pseudotuberculosis* YpIII.

### 3.2 EbSe can alter the polarization status of peritoneal macrophages

As previously reported, intestinal infection related to *Y. pseudotuberculosis* could impair macrophage activity (Seabaugh and Anderson, 2024). To investigate the potential impact of EbSe on macrophage functions, particularly antimicrobial activity, a study was conducted with 48 mice divided into four groups (A-D groups). Groups C and D were gavaged with 2 × 10^9^ CFU/100 μL of YpIII-bioluminescent to construct an acute gastroenteritis models. Further, groups A and C were intraperitoneally injected with DMSO, while groups B and D received intraperitoneal injections of EbSe on days 1- and 3-post-infection, respectively, resulting in groups A (DMSO), B (EbSe), C (YpIII + DMSO), and D (YpIII + EbSe). Then the mice were subjected to peritoneal macrophage extraction on days 4 and 7 post-infection.

**Figure 2:**
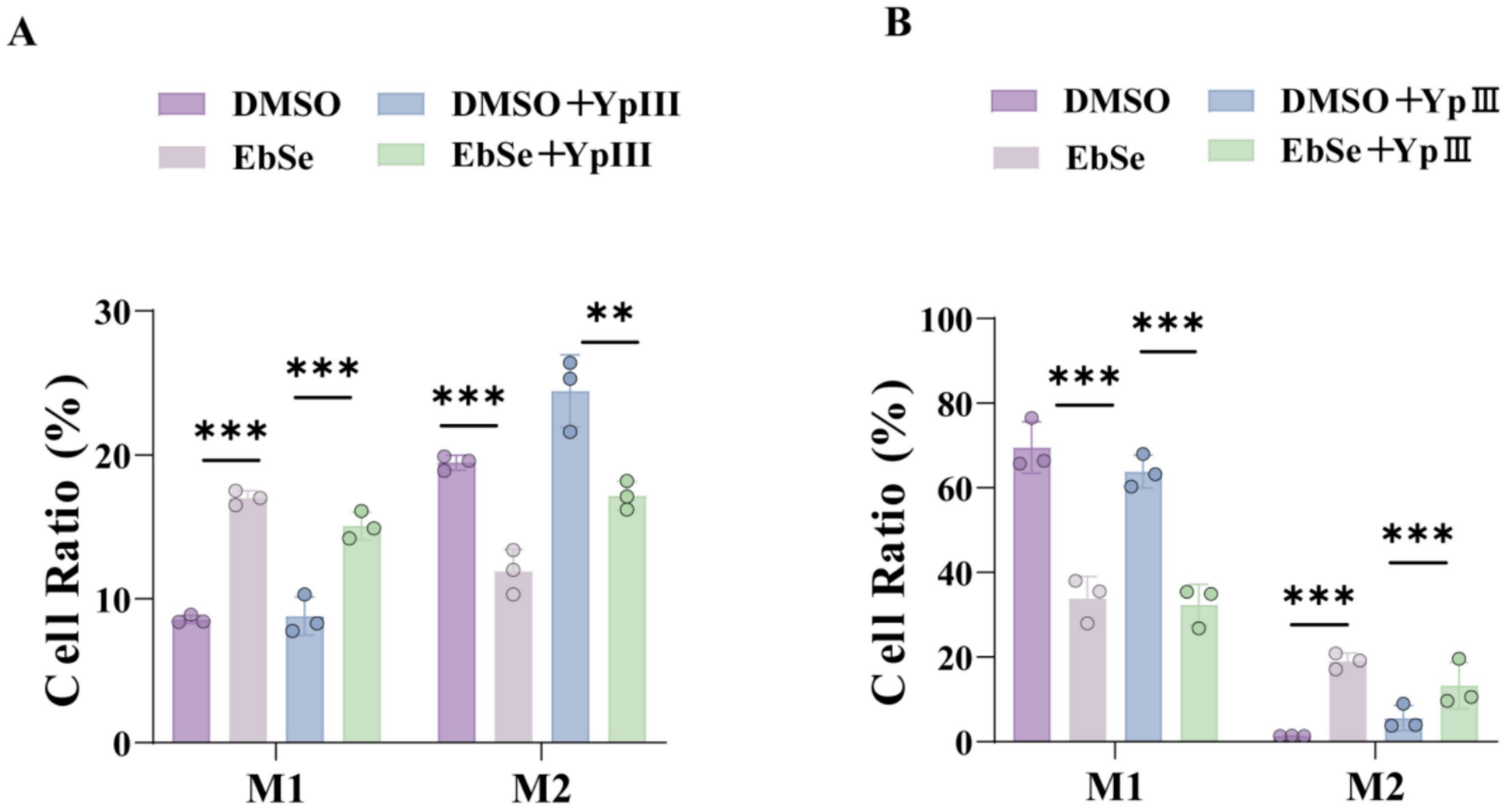
EbSe can alter the polarization status of peritoneal macrophages. *Y. pseudotuberculosis* YpIII-bioluminescent cells were orally administrated into mice and further i.p. administered with EbSe. Peritoneal macrophages were collected for immunofluorescence analyses on days 4 and 7 post-infection. **(A)** The polarization of the peritoneal macrophages on day 4 post-infection (*n* = 3); **(B)** the polarization of the peritoneal macrophages on day 7 post-infection (*n* = 3). *p*-values of < 0.05 were significant. *p < 0.05; **p < 0.01; ***p < 0.001.

The immune-enhancing effect of Ebse was investigated in relation to macrophage polarization by flow cytometry, as previous studies have shown that the polarization of these immune cells plays a crucial role in the immune response and disease progression, following the labeling of mouse peritoneal macrophages with CD68-BV421, CD16/32, CD86-BV510, iNOS, and CD206-PE/Cy7. The results showed that the peritoneal macrophages in the EbSe-treated group have a higher M1 phenotype rate (15.1%) compare to DMSO-treated group (8.8%) on day 4-post-infection, leading to a more intense inflammatory response and realizing the bactericidal effect (Figure 2A). On day 7-post-infection (Figure 2B), the inflammatory response in the EbSe-treated group tended to be reduced compared to DMSO-treated group (5.6%) due to a higher proportion of M2-type cells (13.3%).

### 3.3 EbSe could inhibit *Yersinia pseudotuberculosis* YpIII-induced inflammation in Raw264.7 cells

**Figure 3:**
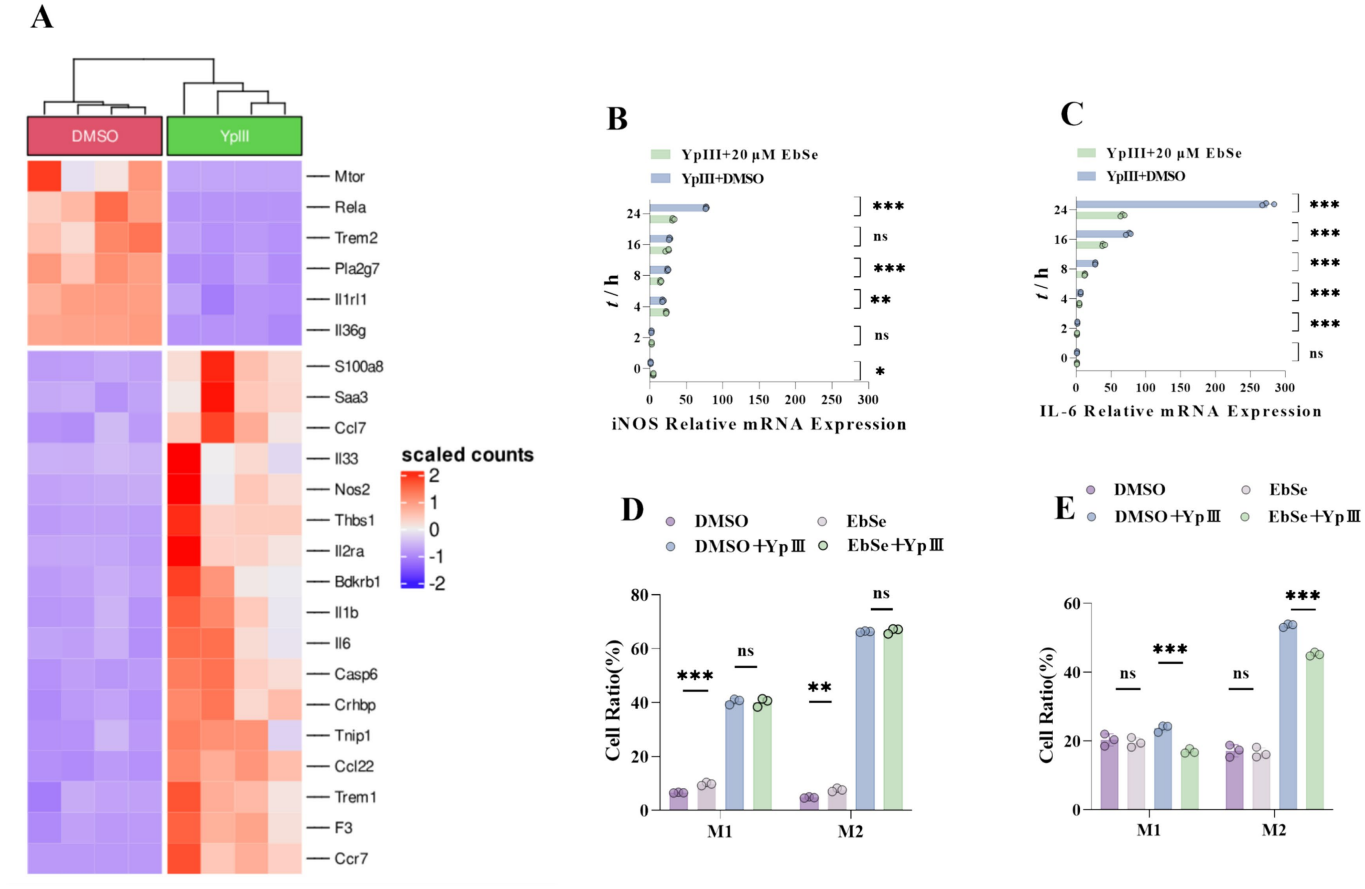
EbSe could inhibit *Y. pseudotuberculosis* YpIII-induced inflammation in Raw264.7 cells. **(A)** RNA-seq analysis of the changes in the expression of inflammatory factors in the transcriptome of EbSe-treated *Y. pseudotuberculosis* YpIII-infected cells; **(B)** RT-qPCR was used to detect the expression level of iNOS, GAPDH was used as an internal reference (*n* = 3); **(C)** RT-qPCR was used to detect the expression level of IL-6 (*n* = 3); **(D)** The polarization status of Raw264.7 cells on 4 h post-infection (*n* = 3); **(E)** The polarization status of Raw264.7 cells on 24 h post- infection (*n* = 3). *p*-values of < 0.05 were significant. *p < 0.05; **p < 0.01; ***p < 0.001.

To explore how EbSe influences macrophages, the transcriptome of *Y. pseudotuberculosis* YpIII-infected Raw264.7 cells was subjected to RNA-seq analysis (Figure 3A). As depicted in Figures 3B,C, EbSe markedly decreased the expression of IL-6 and iNOS, as evidenced by RT-qPCR, thereby effectively mitigating the inflammatory response triggered by YpIII infection. It is demonstrated that a rise in proinflammatory factors can polarize macrophages, inducing their formation into M1-type macrophages (Martinez et al., 2008). However, immunofluorescence investigations revealed that the polarization status of cells in the EbSe-treated group had significantly changed. The results indicated that the counts of M1 and M2 cells in the EbSe-treated group were considerably decreased within 24 h, which largely reduced the inflammatory response (Figures 3D,E). This implies that EbSe takes a part in controlling inflammation in Raw264.7 cells.

### 3.4 EbSe reduces *Yersinia pseudotuberculosis* YpIII-induced cell death in macrophages

**Figure 4:**
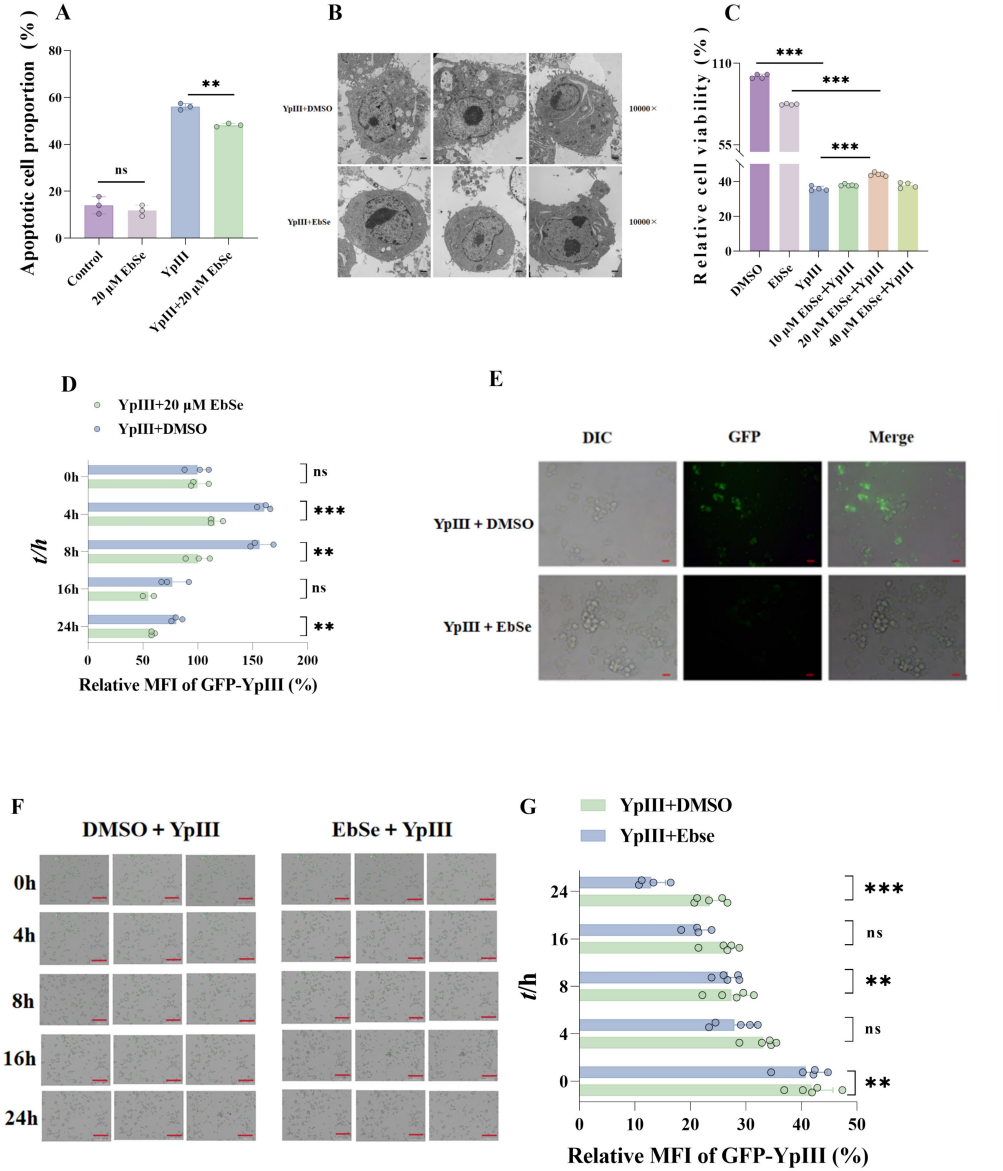
EbSe reduces *Y. pseudotuberculosis* YpIII-induced cell death in macrophages. After *Y. pseudotuberculosis* YpIII infection, Raw264.7 cells were treated for 24 h with an EbSe-containing medium. **(A)** Annexin V-FITC staining was used to quantify the cell apoptosis (*n* = 3); **(B)** transmission electron microscopy was performed to observe the cell morphology; **(C)** cell viability was detected using CCK8 assay (*n* = 3); **(D)** fluorescence activated cell sorting was used to identify the fluorescence intensity of YpIII (*n* = 3); **(E)** fluorescence microscopy analysis was used to identify YpIII fluorescence intensity and cell shape; **(F)** Cytation 5 was used to dynamically monitor the Raw264.7 cells infected with YpⅢ over 24 hours; **(G)** Quantitative analysis of intracellular bacterial fluorescence intensity; Cytation 5 results demonstrate that the EbSe-treated group exhibited reduced bacterial load within Raw264.7 cells while maintaining superior cellular morphology. Scale bar: 200μm. 10 μm is the scale bar. *p*-values of < 0.05 were significant. *p < 0.05; **p < 0.01; ***p < 0.001.

It is reported that *Y. pseudotuberculosis* can disrupt host immune defenses by releasing toxins or activating signaling pathways that lead to cell death (Bergsbaken and Cookson, 2007). To test whether EbSe has any effect on the *Y. pseudotuberculosis* YpIII-infected Raw264.7 cells, flow cytometry was performed. Utilizing Annexin V-PI staining, a method that detects apoptotic cells by identifying the exposure of phosphatidylserine (PS) on the cell membrane, it was observed that the quantity of apoptotic cells rose subsequent to YpIII infection. Conversely, in the EbSe-treated group, a reduction in apoptotic cells was noted (Figure 4A). This staining method involves the use of Annexin V, a protein that binds to PS, and Propidium Iodide (PI), which stains the nuclei of cells that have lost membrane integrity, allowing for the differentiation between early apoptotic cells (Annexin V positive, PI negative) and late apoptotic or necrotic cells (Annexin V and PI positive). Further, transmission electron microscopy (TEM) was utilized to detect morphological changes, revealing that the EbSe-treated group exhibited less cell damage and content leakage (Figure 4B).

Eventually, we found that EbSe could significantly prevent *Y. pseudotuberculosis* YpIII-infected Raw264.7 cells from undergoing cell death (Figure 4C). In addition, to explore the effect of EbSe on the intracellular bacterial load in Raw264.7 cells, the quantities of YpIII-GFP were measured using Cytation 5. The amounts of the YpIII-GFP in EbSe-treated group were a lot less than those of the control group at 4, 8, 16 and 24 h post-infection. And utilizing flow cytometry, a significant reduction in bacterial load was observed in the EbSe-treated group, as well as a decrease in the bacterial count within the cytophagocytic lysozyme, as depicted in Figure 4D. In line with the observations from fluorescence microscopy (Figure 4E), EbSe has been shown to mitigate YpIII-induced cell death by reducing bacterial load in macrophages and maintaining cellular integrity. Further confirmed by Cytation 5 (Figures 4F,G).

It is noteworthy that we have not yet identified markers for necrosis or pyrolysis, which will be the focus of our subsequent experimental phase.

### 3.5 EbSe increases the activity of Txnrd1 and enhances the antioxidant capacity of *Yersinia pseudotuberculosis* YpIII-infected Raw264.7 cells

**Figure 5:**
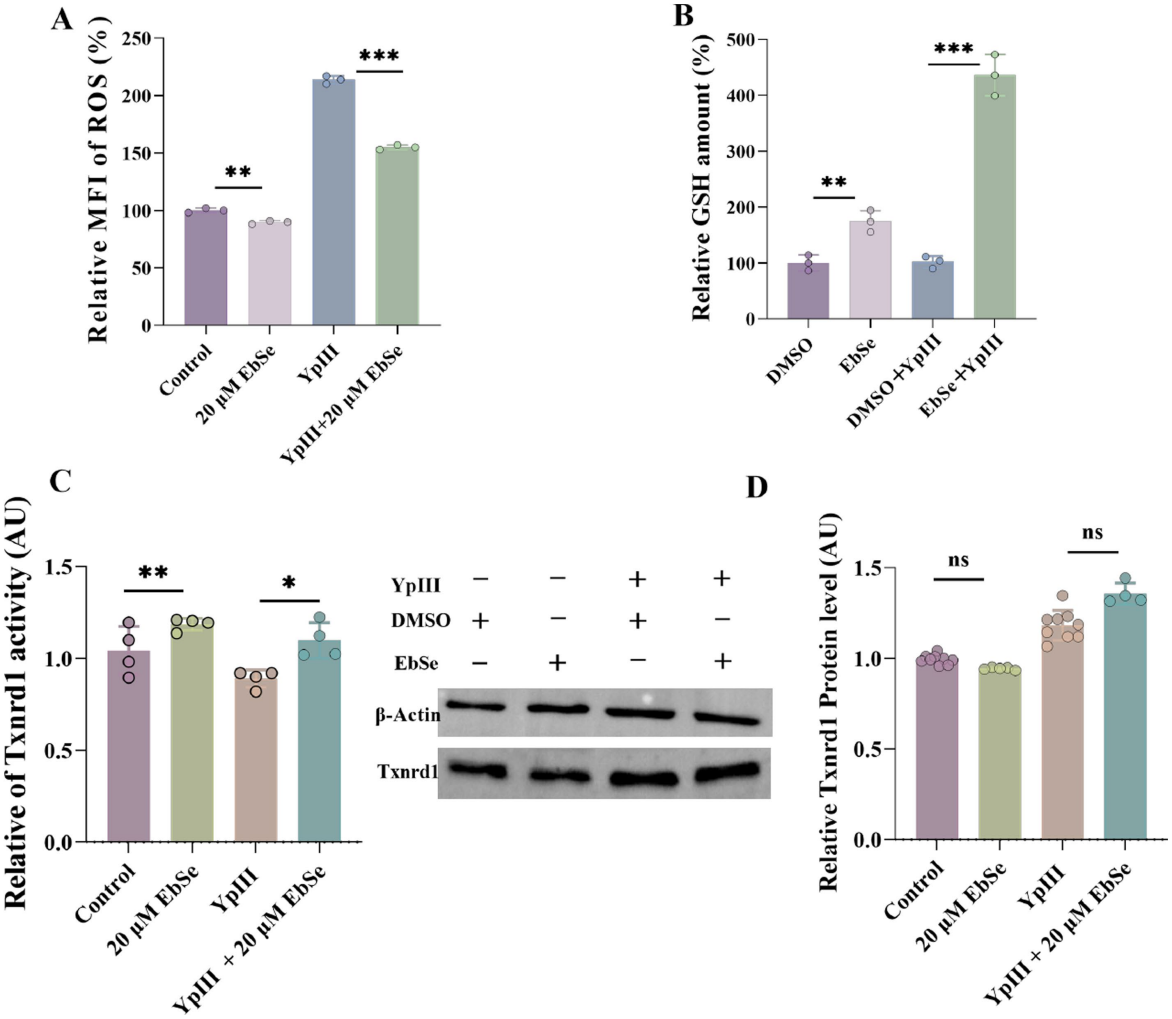
EbSe increases the activity of Txnrd1 and enhanced the antioxidant capacity of *Y. pseudotuberculosis* YpIII-infected Raw264.7 cells. **(A)** Flow cytometry was used to detect intracellular ROS levels (*n* = 3), the relative MFI was calculated based on the control (46.7 ± 0.8), Ebse (42.1 ± 0.7), YpIII (100 ± 1.7), Ebse+YpIII (72.4 ± 1.2); **(B)** Intracellular GSH (*n* = 3) levels were detected by the DTNB assay. The relative GSH amounts were calculated based on DMSO (21.5 ± 4.1), Ebse (37.5 ± 5.8), DMSO+YpIII (22.3 ± 2.7), Ebse+YpIII (93.7 ± 11.3); **(C)** Intracellular Txnrd1 (*n* = 4) activity was detected by the DTNB assay as GSH. **(D)** Western blot was used to detect the intracellular Txnrd1 expression level with *β*-Actin as an internal reference. *p*-values of < 0.05 were significant. *p < 0.05; **p < 0.01; ***p < 0.001.

To ascertain whether EbSe regulates redox homeostasis after *Y. pseudotuberculosis* YpIII infection, two assays were employed. In the initial experiment, Raw264.7 cells were infected and cultured for 24 h, which led to a significant 214% increase in intracellular ROS content. This could be attributed to the cells’ robust response to the infection, as Raw264.7 cells are known for their strong phagocytic capabilities and the release of chemokines upon antigen uptake, potentially influencing the experimental outcome. Following treatment with 20 μM EbSe for an additional 24 h, the intracellular ROS content decreased to 155% (Figure 5A). Consistent with this, in YpIII-infected Raw264.7 cells treated with EbSe, we observed that the GSH level increased from 142 to 473% compared to the control group (Figure 5B). Furthermore, the DTNB assay demonstrated that EbSe enhanced the Txnrd1-reducing activity (Figure 5C), yet not any statistically significant expression levels were determined by Western blot (Figure 5D).

Collectively, all of the above findings suggest that EbSe plays an important role in regulating the level of oxidative stress and antioxidant capacity in cells by reducing intracellular ROS content and enhancing GSH synthesis, thereby enhancing cellular antioxidant defense and potentially impacting the cells’ inflammatory reactions. Considering the demonstrated immunomodulatory effects of EbSe, we suggest that activation of Nrf2-ARE or redox-mediated suppression of NF-κB would serve as the primary focus for subsequent mechanistic investigations.

## 4 Discussion

*Yersinia pseudotuberculosis*, a common food-borne zoonotic pathogen (Kamath et al., 2016), can infect animal hosts through contact transmission, environmental exposure, or via vector intermediaries. Anthropogenic factors, including habitat fragmentation, human activity interference, and increased interspecific contact (Meissner et al., 2022), significantly elevate infection risks in wildlife populations. Similarly, farm animals (such as swine, cattle (Ali et al., 2025), sheep, *etc*.) constitute a high-risk group due to intensive farming practices characterized by high stocking densities, environmental stressors (Domanska-Blicharz et al., 2023), and frequent animal movement. These conditions facilitate the spread of pathogens, resulting in morbidity, production losses, and economic impact on the livestock industry. As the primary cause of bacterial gastrointestinal enteritis in animals, *Y. pseudotuberculosis* infection highlights the urgent need for improved therapeutic strategies. The cytomegalovirus’s ability to replicate intracellularly and evade macrophage-mediated immune surveillance, as elucidated by Moreau et al., 2010, further complicates treatment strategies (Taheri et al., 2016), necessitating the development of novel therapeutic approaches.

As a synthetic organoselenium radical scavenger compound, EbSe has demonstrated significant pharmacological characteristics. Apart from potent antioxidant activity mediated by redox system (Wang J. et al., 2020; Qu et al., 2021; Ren et al., 2018), immune regulation, inhibition of inflammatory (Bjørklund et al., 2022; Yu et al., 2024), and potential anti-depressant treatment (Ramli et al., 2022; Ramli et al., 2024) *etc,* global research has confirmed that EbSe exhibits significant antimicrobial activity against Gram-positive bacterial infections (Felix et al., 2021; Maślanka and Mucha, 2023). While our prior work established EbSe’s promising against Gram-negative bacterial infection (Chen et al., 2022; Dong et al., 2022; Wang P. et al., 2020), the precise pharmacological mechanisms underlying the efficacy are not fully understood, as evidenced by ongoing research efforts in EbSe treating *Y.pseudotuberculosis* infection. Showing no direct antibacterial effect against *Y. pseudotuberculosis in vitro* (Dong et al., 2022), EbSe managed to prevent mice from developing gastroenteritis, which suggests that EbSe may possess immune-enhancing properties applicable in HDT strategy.

Based on this, the current study utilizes a *Y. pseudotuberculosis*-induced acute gastroenteritis murine model to verify EbSe’s *in vivo* efficacy. Subsequent analyses demonstrate that EbSe increases the overall survival of mice with an acute infection by *Y. pseudotuberculosis* (Figure 1B). EbSe significantly reduces the bacterial load, as evidenced by the bioluminescence overflux (Figures 1C,D). To focus on the macrophages against infection (Murray, 2017), the observed polarization shift in peritoneal macrophages implies that EbSe could potentially promote the M1 phenotype (Figure 2A) on day 4-post-infection, which is commonly associated with bacterial clearance by inducing T cells but also produce proinflammatory chemicals and oxygen free radicals (Wang et al., 2014; Podinovskaia et al., 2013), thus promoting a more favorable immune response (Martinez et al., 2008). In contrast, the transition to M2 macrophages on day 7-post-infection suggests a flexible response where the inflammatory reaction subsequently decreases to aid in reducing tissue damage (Figure 2B). Moreover, the ability of EbSe to prevent *Y. pseudotuberculosis*-induced cell death (Figures 4A–E) in Raw264.7 cells highlights the cytoprotective properties of this compound, align with previous studies indicating that enhancing macrophage survival can improve pathogen clearance and bolster the overall immune system. Its anti-inflammatory properties are further demonstrated by the RNA-seq that shows a decreased expression of pro-inflammatory cytokines including IL-6 and EbSe resulted in changes in iNOS expression (Figures 3A–C). Given that excessive inflammation can lead to tissue damage and impaired immune function, it is essential to regulate inflammatory pathways. Recent research has shown that therapies promoting macrophage polarization toward the M2 phenotype can effectively reduce inflammatory responses across various conditions, as evidenced by Wynn et al., 2013. Our findings indicate that the EbSe markedly alters the cellular redox equilibrium in YpIII-infected cells, potentially by decreasing ROS levels and increasing GSH contents, which is attributed to EbSe’s ability to not only reduce oxidative stress but to enhance the antioxidant defense systems within cells, as evidenced by the increased Txnrd1-reduction activity (Figures 5A–D), indicating that the ROS levels will decrease with the bacterial antioxidant property which led to the reduction of IL-6/iNOS levels. Recent advancement (Stoolman et al., 2025) also suggests that optimizing the redox state of immune cells can significantly bolster their efficacy in combating infections and diseases. Hence, EbSe (Lu et al., 2021) introduces a novel HDT approach that effectively combats Gram-negative pathogens via immunomodulatory mechanisms, by bolstering the host’s antioxidant defenses, modulating macrophage polarization, and enhancing cell viability.

Compared to other immune-enhancing agents, including grape seed extract (Gupta et al., 2020), mushroom extract (Nasri et al., 2025), Milkvetch Root (Guo et al., 2016), and polysaccharide (Zhang et al., 2022), or HDT candidates such as cytokine modulators and metabolic reprogrammers (Yahfoufi et al., 2018), EbSe or EbSe-derived compounds stand out as more excellent candidates for its safety profile and tolerability in humans supported by clinical investigations (e.g., NCT03013400), enabling its evaluation for conditions like stroke and Alzheimer’s disease (AD) which has entered Phase III and Phase II clinical trials. To advance the translation to clinical veterinary applications, future research must address the regulatory and formulation considerations, and develop scalable EbSe-based formulations that are efficient across species to ensure consistent performance. Worth noting that the current research has several limitations, we expect to expand our range of Gram-negative bacterial infection animal models and cell lines, and evaluate the relations between EbSe and the specific pathways as Nrf2, NF-κB and MAPKs. Furthermore, it is imperative to conduct thorough assessments of the long-term safety and dose–response profiles of EbSe in animal models to ensure readiness for clinical trials.

Taken together, we propose EbSe as a promising host-directed therapy (HDT) candidate against *Y. pseudotuberculosis* infection through host immune potentiation that regulates oxidative stress in macrophages to enhance their antimicrobial activity, offering a novel strategy to combat bacterial infections.

## 5 Conclusion

Collectively, our previous studies have demonstrated that EbSe significantly alleviated YpIII infection in both *in vivo* acute gastroenteritis murine models and *in vitro* Raw264.7 cell cultures, with its therapeutic efficacy mechanistically attributed to modulation regarding macrophage polarization, and it suppresses excessive inflammation, cell death, and enhances antioxidant defense by reducing ROS, elevating GSH levels, and increasing the activity of Txnrd1.

Moving forward, further mechanistic investigations are also warranted to uncover the additional pathways involved in its anti-YpIII infection effects, while systematic dosage optimization and long-term safety evaluations will be essential to validate its clinical feasibility. Given the high prevalence of YpIII across diverse animals, exploring the potential veterinary applications of Ebse are also pivotal for expanding the translational impact of this promising HDT approach in the prevention and treatment of bacterial pathogens through the augmentation of host immunity.

## Data Availability

The datasets presented in this study can be found in online repositories. The names of the repository/repositories and accession number(s) can be found here: https://www.ncbi.nlm.nih.gov/, accession number PRJNA1406460.
